# Insights into the Structure of the Vip3Aa Insecticidal Protein by Protease Digestion Analysis

**DOI:** 10.3390/toxins9040131

**Published:** 2017-04-07

**Authors:** Yolanda Bel, Núria Banyuls, Maissa Chakroun, Baltasar Escriche, Juan Ferré

**Affiliations:** ERI BIOTECMED and Department of Genetics, Universitat de València, Dr. Moliner, 50, BURJASSOT, 46100 Valencia, Spain; yolanda.bel@uv.es (Y.B.); nuria.banyuls@uv.es (N.B.); chakrounmaissa7@gmail.com (M.C.); baltasar.escriche@uv.es (B.E.)

**Keywords:** Vip proteins, bacterial secreted proteins, toxin activation, proteolytic activation, trypsin inhibitors, *Bacillus thuringiensis*, SDS-PAGE artefact, protease stability

## Abstract

Vip3 proteins are secretable proteins from *Bacillus thuringiensis* whose mode of action is still poorly understood. In this study, the activation process for Vip3 proteins was closely examined in order to better understand the Vip3Aa protein stability and to shed light on its structure. The Vip3Aa protoxin (of 89 kDa) was treated with trypsin at concentrations from 1:100 to 120:100 (trypsin:Vip3A, *w*:*w*). If the action of trypsin was not properly neutralized, the results of SDS-PAGE analysis (as well as those with *Agrotis ipsilon* midgut juice) equivocally indicated that the protoxin could be completely processed. However, when the proteolytic reaction was efficiently stopped, it was revealed that the protoxin was only cleaved at a primary cleavage site, regardless of the amount of trypsin used. The 66 kDa and the 19 kDa peptides generated by the proteases co-eluted after gel filtration chromatography, indicating that they remain together after cleavage. The 66 kDa fragment was found to be extremely resistant to proteases. The trypsin treatment of the protoxin in the presence of SDS revealed the presence of secondary cleavage sites at S-509, and presumably at T-466 and V-372, rendering C-terminal fragments of approximately 29, 32, and 42 kDa, respectively. The fact that the predicted secondary structure of the Vip3Aa protein shows a cluster of beta sheets in the C-terminal region of the protein might be the reason behind the higher stability to proteases compared to the rest of the protein, which is mainly composed of alpha helices.

## 1. Introduction

*Bacillus thuringiensis* (Bt) is a ubiquitous Gram-positive sporulating bacterium that produces several entomopathogenic proteins. The proteins that have received more attention, and are thus the best known, are the δ-endotoxins (Cry and Cyt toxins), produced as parasporal crystalline inclusions during the stationary phase of growth. Other proteins associated with insecticidal activity, including the Vip proteins, are secreted into the medium during the vegetative growth phase [1]. Some Cry and Vip proteins (such as Vip3A proteins) show high insecticidal activity against a wide range of insect species (for a review, see van Frankenhuyzen 2009 [2] for Cry proteins, and Chakroun et al. 2016 [3] for Vip proteins), and the genes encoding them have been transferred to crop plants to protect them against insect pests.

Vip3 proteins do not share sequence homology with other Bt insecticidal toxins and their 3D structure is yet unknown. The proposed mode of action of Vip3A proteins shares some similarities with that of the Cry proteins, in that both undergo activation (proteolytic processing) in the insect midgut, bind to receptors on the surface of the midgut cells, and, finally, make pores that lead to cell lysis, septicemia, and eventually death of the insect [3,4,5,6,7]. The molecular processes behind this cascade of events are still unclear for Vip3A proteins and differ from those of the Cry proteins. The binding sites of the Vip3A proteins in the midgut are different from those described for the Cry proteins [8,9,10,11,12], and the cell pores formed are structurally and functionally different [5]. The high insecticidal activity of the Vip3A proteins, along with the differences in the mode of action with Cry proteins, has prompted their use in crop protection and pest management. Some Bt-cotton and Bt-corn varieties combine the expression of Vip3Aa with one or more Cry proteins [13].

Vip3A proteins (MW about 89 kDa) have an N-terminal signal sequence that, unlike most secreted proteins, is not processed when the protein is delivered to the media [14]. Once in the midgut of the insect, as a first step in the mode of action, the Vip3A proteins are activated. The activation is necessary since the full length Vip3Aa is unable to form pores in vitro [5], and differences in the rate of activation have been related with differences in susceptibility amongst lepidopteran species [15,16,17]. Furthermore, reduced protease activity has been found in a Vip3Aa-resistant strain of *Helicoverpa armigera* (Lepidoptera: Noctuidae) [18], and it has been proposed as the mechanism of resistance in a Vip3Aa resistant strain of *Spodoptera litura* (Lepidoptera: Noctuidae) [19].

The activation of the Vip3Aa protein by the insect midgut juice (MJ) was described soon after its discovery. Incubation of Vip3Aa with insect MJ led to four major proteolysis products of about 62, 45, 33, and 22 kDa [20]. Similar patterns of proteolysis (with a band of about 65 kDa and several other bands of lower molecular weight) have been observed by other authors with MJ from many insect species [5,8,9,15,21,22,23]. Similarly, the in vitro activation of Vip3A proteins with trypsin produces a major fragment of about 62–65 kDa, along with other fragments, mainly one of about 20 kDa that would correspond to the N-terminal region [24]. Although the 33 kDa fragment was proposed to be the minimum toxic fragment after proteolysis [24], further studies have led to the 62–65 kDa protein being considered the protease resistant core and the active form of the protein [5,6,8,9,15,16,22,25,26,27]. However, some studies on the stability of Vip3A proteins to proteases seemed to show that the 62–65 kDa core was not stable, as revealed by SDS-PAGE, since the 62–65 kDa fragment was processed to smaller fragments when the concentration of proteases was increased [22,28,29].

In the present work, the activation process for Vip3 proteins was closely examined in order to better understand the Vip3Aa protein stability and to shed light on its structure. In our hands, the SDS-PAGE analysis of the Vip3Aa protein processed at high concentrations of trypsin (or MJ) indicated an apparently fast and complete degradation of the protein. However, when the proteolytic reaction was efficiently stopped, it was revealed that the protoxin had been only cleaved at a primary cleavage site, regardless of the amount of proteases used, generating two bands of 66 kDa and 19 kDa. These findings are important for the interpretation of many published results in which Vip3A processing is shown. Finally, the trypsin treatment of the protoxin in the presence of sodium dodecyl sulfate (SDS) revealed the presence of secondary cleavage sites that have allowed us to propose a relationship between the predicted secondary structure of the protein and its stability.

## 2. Results

### 2.1. Stability of Vip3Aa Protoxin to Trypsin Processing

**Figure 1:**
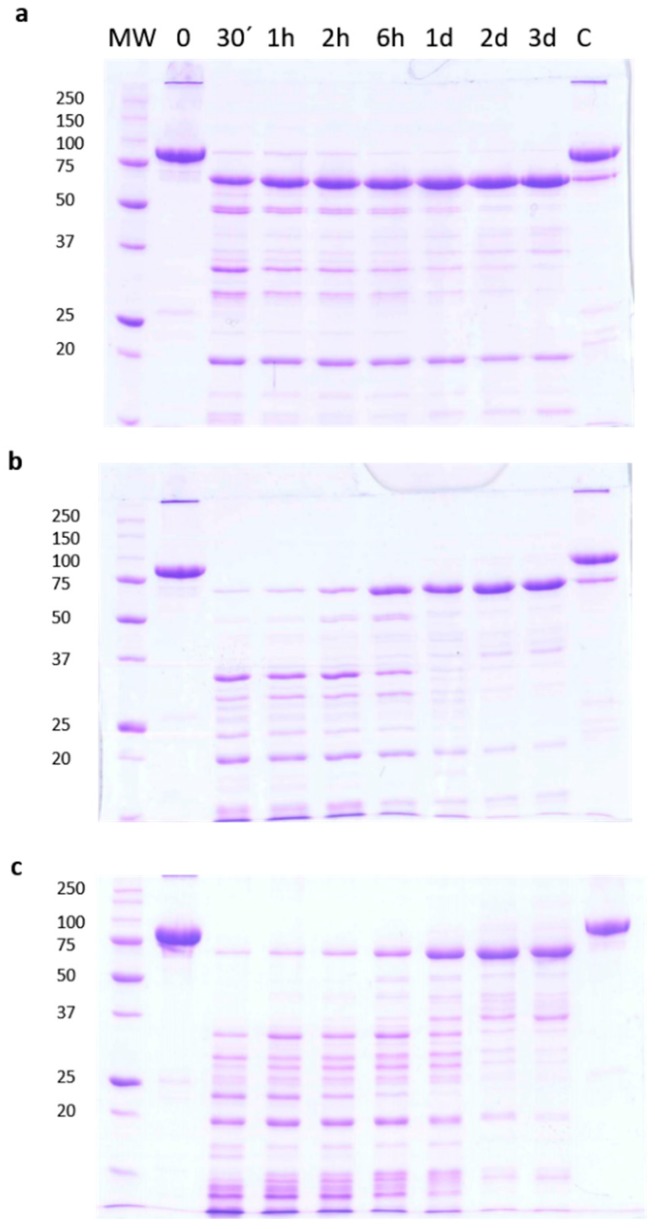
Time course of trypsin processing of Vip3Aa, as revealed by SDS-PAGE. Reactions were performed at 30 °C at different concentrations of trypsin in Tris-HCl buffer. The ratios of trypsin:Vip3A (*w*:*w*) were: (**a**) 1:100, (**b**) 24:100, and (**c**) 120:100. Aliquots were withdrawn at different times, as shown at the top of each lane. Molecular weight markers (MW) are indicated in kDa. C = protoxin after 3 days of incubation at 30 °C.

Trypsin treatment of the Vip3Aa protein in Tris-HCl buffer (pH 8.6), at 1:100 trypsin:Vip3A (*w*:*w*) for 30 min (corresponding to the conditions used in most studies) rendered a major band of around 66 kDa (usually identified as the Vip activated toxin), as well as other smaller bands of about 42, 32, 29, and 19 kDa (Figure 1a). Whereas the concentration of the smaller bands decreased with the incubation time, the 66 kDa band seemed to become more intense as the incubation proceeded. To confirm this observation, the experiment was repeated using different ratios of trypsin:Vip3A (24:100 and 120:100, *w*:*w*). In these conditions, the accumulation with time of the 66 kDa band became even more evident, and at the same time the smaller bands eventually disappeared (Figure 1b,c). This phenomenon was also observed when trypsin digestion was performed at a lower temperature (4 °C), as well as when the Tris buffer was substituted by carbonate buffer (pH 10.5) (data not shown).

**Figure 2:**
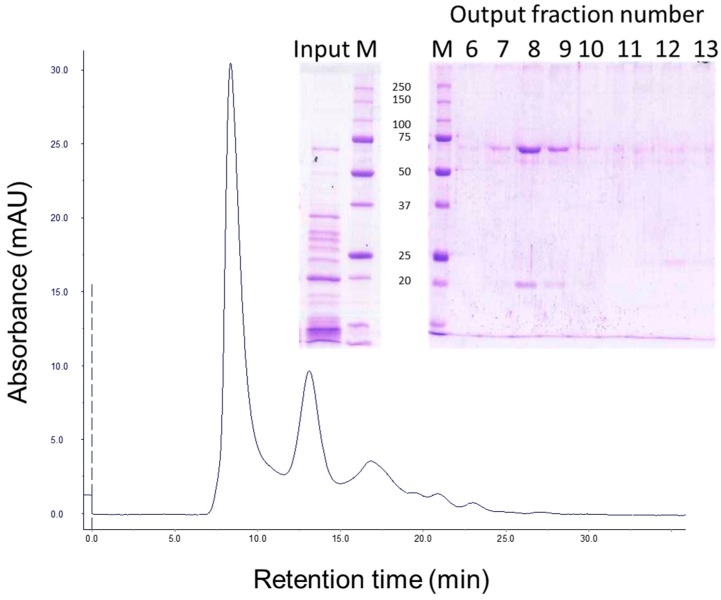
Gel filtration chromatography of Vip3Aa treated with trypsin. Vip3Aa was incubated with trypsin (24:100 trypsin:Vip3A, *w*:*w*) for 30 min (“Input” in figure inset). The sample was loaded into a Superdex-75 10/300 GL column and elution fractions (1 mL each) were analysed by SDS-PAGE (“Output” in figure inset). Molecular weight markers (M) are indicated in kDa.

Gel filtration chromatography of the 30 min processed Vip3Aa protein (with 24:100 trypsin:Vip3Aa) showed a main peak at 8.3 min, corresponding to the void volume of the column (the exclusion limit for globular proteins of this column was 100 kDa), and a peak at 13.0 min (of around 23 kDa by SDS-PAGE), which corresponded to trypsin (Figure 2, fraction 12). SDS-PAGE analysis of the fractions containing the first peak showed two main bands, one of 66 kDa and the other of 19 kDa (Figure 2, lanes 8 and 9 in inset), suggesting that the band pattern obtained at short incubation times in Figure 1 is the result of the trypsin acting on Vip3Aa while in the loading buffer. Furthermore, the presence of both polypeptides in these fractions indicated that, once cleaved, the 19 kDa peptide remains bound to the 66 kDa polypeptide. The elution of the cleaved Vip3Aa protein in a peak corresponding to a molecular size larger than 100 kDa indicates that either the cleaved Vip3Aa protein occurs in solution either as an oligomeric protein, or that it adopts a non-globular shape.

### 2.2. Checking the Efficiency of Protease Inhibitors or High Concentration Urea on Stopping the Trypsin Action

**Figure 3:**
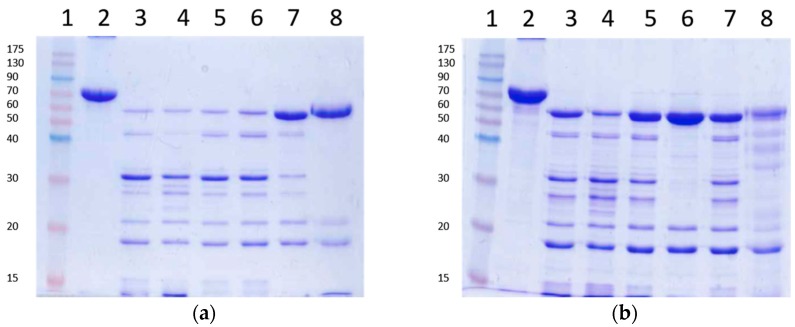
Trypsin digestion of Vip3Aa using inhibitors to stop the reaction. The reactions were stopped with the addition of irreversible trypsin protease inhibitors or urea. Loading buffer was added either immediately (**a**) or 10 min after addition of the inhibitors or the urea (**b**) and the samples heated and subjected to SDS-PAGE. Lanes 1: molecular weight markers; lanes 2: untreated protoxin; lanes 3: control with no inhibitors; lanes 4: 1 mM PMSF; lanes 5: 0.1 mM TLCK; lanes 6: 1 mM AEBSF; lanes 7: 10 mM E64; lanes 8: 8 M urea. Molecular weight markers are indicated in kDa.

Several irreversible trypsin protease inhibitors (PMSF, TLCK, AEBSF, E64), as well as a denaturant agent (8 M urea), were used to stop the action of trypsin upon Vip3Aa prior to SDS-PAGE. The protease inhibitors or the urea were added after 30 min of Vip3Aa incubation with trypsin (24:100 trypsin:Vip3Aa, *w*:*w*). The loading buffer was added either immediately or 10 min after addition of the inhibitors or the urea, and then the samples were heated for SDS-PAGE. The inhibitors were used at the highest concentration recommended in the literature. The results showed that none of the tested trypsin inhibitors stopped the action of trypsin when the loading buffer was added just after the addition of the inhibitors (Figure 3a). In these conditions, only E64 was able to partially stop the reaction. However, if the addition of loading buffer was delayed by 10 min, the AEBSF inhibitor was able to completely stop the trypsin action, rendering a pattern in SDS-PAGE of three bands: the 66 and 19 kDa peptides derived from the Vip3Aa protoxin, and a 23 kDa band corresponding to trypsin (lane 6 in Figure 3b). Unexpectedly, after preincubation, E64 performed worse than in the previous conditions. These experiments were replicated using a lower rate of trypsin:Vip3Aa (10:100, *w*:*w*) and a concentration of inhibitors 10 times higher; the results did not change (data not shown). Therefore, except for AEBSF, the rest of the inhibitors tested were not able to completely inhibit the activity of trypsin, even when used at very high concentrations. On the other hand, 8 M urea was very efficient at stopping the action of trypsin, rendering a profile similar to that obtained with AEBSF (Figure 3a). Some minor bands could be observed after a 10 min preincubation in the presence of urea (Figure 3b), probably because a small fraction of Vip3Aa became digested by trypsin while becoming denatured.

Taken together, the above experiments show that when the trypsin activity is completely inhibited (by either AEBSF or 8 M urea) before SDS-PAGE, only two tryptic fragments are generated—of 66 kDa and 19 kDa—even at extremely high concentrations of trypsin (24:100 trypsin:Vip3A, *w*:*w*). Thus, Vip3Aa has just one primary cleavage site under native conditions, as opposed to the several secondary cleavage sites revealed under denaturing conditions.

### 2.3. Analysis of the Biological Activity of the Trypsin-Treated Vip3A Protein

To determine whether the samples treated with high concentrations of trypsin (24:100 trypsin:Vip3A, *w*:*w*) for different times (and with very different SDS-PAGE profiles) differed in their insecticidal activity, bioassays were performed with *Agrotis ipsilon* (Lepidoptera: Noctuidae) neonate larvae. The results showed that the Vip3Aa samples treated for either 30 min or 3–4 days retained insecticidal activity similar to the unprocessed protein (Table 1). Since the 19 kDa fragment disappears with time, the results suggest that this fragment is not essential for toxicity.

### 2.4. Vip3Aa Processing by Trypsin in the Presence of SDS and β-Mercaptoethanol

**Figure 4:**
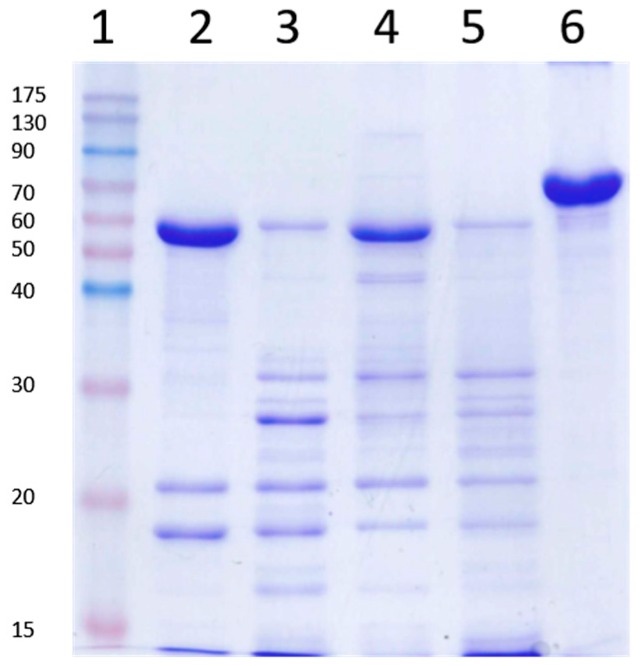
Trypsin processing of Vip3Aa in the presence of sodium dodecyl sulfate (SDS) and β-mercaptoethanol. Vip3Aa was treated with trypsin (24:100 trypsin:Vip3A, *w*:*w*) with or without SDS or β-mercaptoethanol, incubated for 30 min at 30 °C and subjected to SDS-PAGE. Lane 1: molecular weight markers; lane 2: control without SDS or β-mercaptoethanol; lane 3: with SDS; lane 4: with β-mercaptoethanol; lane 5: with SDS and β-mercaptoethanol; lane 6: Vip3Aa without trypsin (protoxin). All reactions were stopped with 1 mM AEBSF followed by 10 min incubation at room temperature (RT). Molecular weight markers (kDa) are indicated in the left. The band of around 23 kDa corresponds to trypsin.

Since the SDS-PAGE loading buffer contains SDS and β-mercaptoehanol, experiments were performed in which the Vip3Aa protoxin was incubated with trypsin (for 30 min at a 24:100 trypsin:Vip3A, *w*:*w*), in the presence of SDS and/or β-mercaptoehanol (at the same concentrations that they would be once the loading buffer is added to the sample). The reactions were stopped with 1 mM AEBSF, let stand for 10 min at RT, and processed for SDS-PAGE as usual. The results showed that the presence of SDS reproduced the same band pattern observed when the trypsin reaction is not stopped with protease inhibitors before SDS-PAGE (Figure 4), evidencing that bands other than 19 and 66 kDa appear by the action of trypsin in the presence of SDS on secondary cleavage sites.

### 2.5. Identification of Peptides Generated by the Trypsin Treatment

Molecular weight analysis by MALDI TOF/TOF of the trypsin-treated Vip3Aa protein incubated for 3 days (at 24:100 trypsin:Vip3A, *w*:*w*) revealed peaks of mass/charge of 66539, 33246, and 22169, which corresponded to a 66 kDa polypeptide (66.539 kDa exact molecular weight) with one, two, and three charges, respectively (see Supplementary Figure S1).

The tryptic peptide mass fingerprint was obtained for the main bands after trypsin treatment, followed by SDS-PAGE. The results allowed us to putatively identify the SDS-PAGE bands based on the Vip3Aa16 sequence (GenBank Acc. No. AAW65132.1). Unfortunately, this type of analysis does not always allow one to pinpoint the exact N- and C-terminus of the peptides, since some tryptic fragments which are generated are either too small or too large to allow detection. The fingerprinting results unambiguously indicated that the band of 66 kDa consisted of a polypeptide starting at amino acid residue D-199 and ending at the C-terminus of the protein (amino acid K-789). The fingerprint of the band of approximately 19 kDa matched with sequences from the N-terminal region of the protein, starting at the N-terminus and ending at either K-195, K-197, or K-198 (most likely the latter). The bands of approximately 42, 32, and 29 kDa all gave matches with the C-terminal part of the protein. The start residue of the band of 29 kDa was confirmed by Edman’s degradation, and was identified as S-509; the last residue of this band coincided with the protein C-terminus. The bands of 42 and 32 kDa yielded tryptic fragments matching the region covered by the band of 29 kDa, indicating that they are larger versions of the 29 kDa fragment.

### 2.6. Stability of Vip3Aa Protoxin to A. ipsilon Midgut Juice

**Figure 5:**
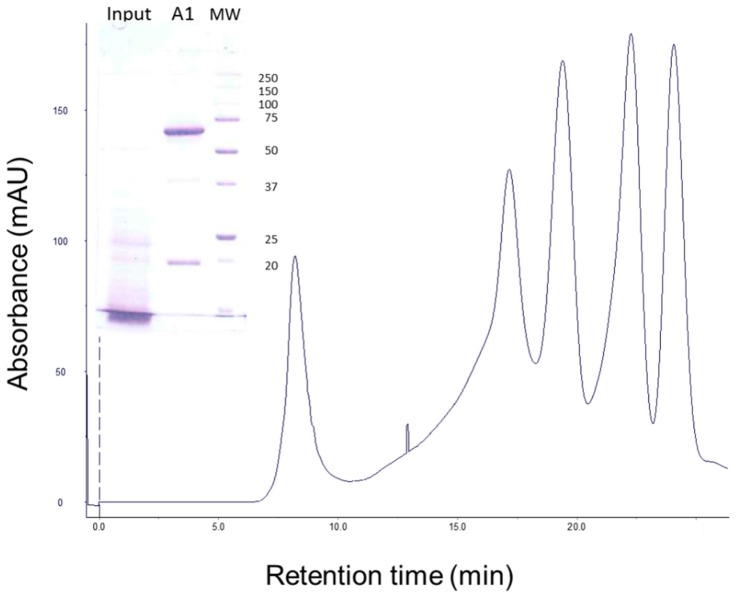
Gel filtration chromatography of Vip3Aa treated with *A. ipsilon* midgut juice (MJ). Vip3Aa was incubated with 40:100 MJ:protein (*w*:*w*) for 1 h (“Input” in figure inset). The sample was loaded into the column and the elution fractions (1 mL each) were analysed by SDS-PAGE. The protein profile of the output (fraction A1) is shown in the figure inset. Molecular weight markers (MW) are indicated on the right in kDa.

When the Vip3Aa protein, apparently degraded after treatment with a very high concentration of MJ (40:100, MJ:Vip3A, *w*:*w*) (Figure 5, lane of Input in inset), was subjected to gel filtration chromatography, the Vip3Aa protein eluted as a single main peak at 8.35 min, along with several other peaks associated with the MJ (Figure 5). SDS-PAGE of the fractions that contained the 8.35 min peak showed two main bands, one of 66 kDa and the other of 19 kDa (Figure 5, lane A1 in inset). This result confirmed the existence of a core of 66 kDa extremely stable to MJ proteases, and showed that the apparent degradation of Vip3Aa at high concentrations of MJ, as observed by SDS-PAGE, was an artefact.

## 3. Discussion

Since the discovery of Vip3 proteins, the mode of action on susceptible insects has been assimilated to that of the much better known Cry proteins. Although there are important differences, especially at the membrane target sites, the main steps have been mirrored in those of the Cry proteins, including the activation of the protein by proteases in the midgut. Therefore, the full length Vip3Aa protein, or protoxin, is activated in the insect midgut and produces a protease resistant core, the one identified as the 62–66 kDa fragment. This fragment has been proposed to be the one crossing the peritrophic membrane and binding to specific sites in the epithelial membrane. However, this model has been challenged by results which have pointed out that the 62–66 kDa fragment is not as resistant to proteases as originally thought. Although the 62–66 kDa fragment appears as the main proteolysis band in SDS-PAGE when the concentration of trypsin or midgut juice is low, it is no longer the main band in SDS-PAGE gels when higher concentrations of proteases are used [22,28,29]. Many other studies have shown the apparent instability of the 62–66 kDa fragment [5,6,8,15,23], and similar results have been obtained with the closely related Vip3Ca protein [30].

Our results show that, when exposed to trypsin or MJ proteases (even at very high concentrations), the Vip3Aa protoxin is cleaved at a primary cleavage site—between amino acids 198 and 199—rendering two fragments of 19 kDa and 66 kDa which are stable to further processing. The stability to further processing of the 66 kDa core is extremely high: it withstands concentrations as high as 120:100 trypsin:Vip3Aa and 40:100 *A. ipsilon* MJ:Vip3Aa, no matter the incubation time, a situation close to that encountered in vivo when the protoxin is ingested by the larva.

The apparent degradation of the 66 kDa fragment at high concentrations of trypsin or MJ is due to the action of the proteases upon addition of SDS with the loading buffer. This is inferred from the results when the sample is subjected to gel filtration chromatography and trypsin is separated from the activated Vip3Aa prior to SDS-PAGE analysis (Figure 2), or when the reaction is properly stopped (Figure 3). As clearly shown in Figure 4, trypsin can act on the target protein even in the presence of this concentration of denaturant; presumably because Vip3Aa unfolds before trypsin is inactivated, making available less accessible cleavage sites. The unexpected increase of the 66 kDa band with time at high concentrations of trypsin (Figure 1) can be explained as follows: since trypsin autodigests, the shorter the incubation time of the Vip3Aa protein with trypsin, then the higher the trypsin concentration still present in the sample; and thus, the more efficient processing of the 66 kDa peptide by trypsin under denaturing conditions.

Gel filtration chromatography shows that the proteolytically processed Vip3Aa protein elutes as a high molecular mass protein. The SDS-PAGE analysis of the elution peak shows two bands of 66 kDa and 19 kDa, which indicates that these two molecules remain associated under native conditions, as was previously reported [30]. The elution of the processed protein from the gel filtration column as a high molecular mass protein (the exclusion limit of the column is 100 kDa) is in agreement with a recent study that shows that the trypsin-activated Vip3Aa protein aggregates in solution to form an oligomer or because the protein may adopt a non-globular shape [31]. Other examples are known of proteins where, after activation, the two main fragments remain together and co-elute chromatographically. The MJ-activated Cry8Da protein (64 kDa) can be further digested, giving two bands of 54 kDa and 8 kDa by SDS-PAGE, but which elute together by gel filtration chromatography [32]. Also, Cry4A is cleaved into two fragments of 20 kDa and 45 kDa by protease activation that cannot be separated by gel filtration chromatography [33]. Two fragments of 55 kDa and 8–11 kDa, which cannot be separated by size exclusion chromatography, are also obtained during the activation of the coleopteran active Cry3Aa protein [34].

The role of the 19 kDa in the toxicity of the activated Vip3Aa is controversial. While Li et al. [28] found that deletion of the N-terminal first 189 amino acids abolished the insecticidal activity of a chimeric Vip3AcAa protein, Gayen et al. [35] found that a Vip3Aa deletion mutant lacking the first 200 amino acids of the protein not only did not abolish the activity, but it slightly enhanced it against the three insect species tested, and that the expression of this deleted protein in tobacco plants provided even higher plant protection against several feeding insects than the expression of the wild type protein [36]. It is possible that the discrepancy between these two studies is due to the method used for the expression and purification of the deletion mutants, if not to the different Vip3A proteins used. Other studies with smaller deletions also gave contradictory results [37,38,39]. According to our results, the presence of the 19 kDa fragment in the toxicity seems not to be essential, since similar insecticidal activities were found for the samples incubated at 24:100 trypsin: Vip3Aa for 30 min or 3 days (Table 1), with the latter almost lacking the 19 kDa fragment (Figure 1).

An unexpected result from this study is the difficulty in completely terminating the reaction of Vip3Aa with trypsin. Even the highest concentration of inhibitors recommended by the suppliers was unable to stop the reaction, except for AEBSF, and this only after incubation for 10 min. This lack of efficacy, along with the susceptibility of Vip3Aa to SDS unfolding, is responsible for the degradation patterns of Vip3 proteins obtained when analyzed by SDS-PAGE. In the light of our results, all previous data on Vip3 proteins proteolysis, either by commercial proteases or by MJ, should be revised, since most reported band patterns would reflect the susceptibility of the Vip3 protein under denaturing conditions. This would more severely affect those experiments performed at high concentrations of either trypsin or insect midgut juice. It is very likely that the lack of termination of the trypsin reaction would be the reason why the 33 kDa fragment was originally proposed to be the minimum toxic fragment after proteolysis [24].

**Figure 6:**
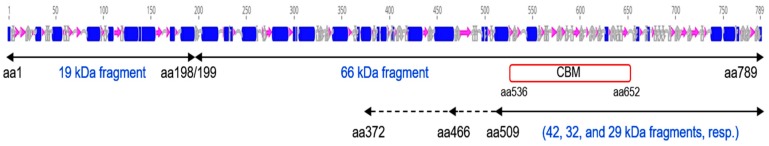
Schematic representation of the Vip3Aa secondary structure and identification of the peptides generated by trypsin digestion of Vip3Aa. Alfa helices are represented as blue cylinders, beta sheets are represented as purple arrows, and turns are represented in gray. CBM = Predicted Carbohydrate Binding Motif. Black arrows under the secondary structure represent the polypeptides identified by Mass fingerprinting.

No 3D structure of any Vip3 protein has yet been resolved. To shed light on the Vip3Aa structure, we have exploited the susceptibility of this protein to trypsin digestion under denaturing conditions (i.e., in the presence of SDS) to uncover secondary trypsin sites. The main bands obtained upon trypsin treatment of Vip3Aa in the presence of SDS were analyzed by MALDI TOF/TOF and the tryptic fragments were identified based on the Vip3Aa16 sequence (GenBank Acc. No. AAW65132.1). The analysis provided the exact molecular weight of the main fragment (66.539 kDa) and identified the 19 kDa fragment as the N-terminal fragment generated by the primary cleavage site. Secondary cleavage sites yielded the bands of approximately 42, 32, and 29 kDa, whose tryptic fragments gave matches with the C-terminal part of the protein, suggesting that this region is the most stable region of the 66 kDa core. Interestingly, the predicted secondary structure of the Vip3Aa protein shows a cluster of beta sheets in the C-terminal region of the protein (where the putative carbohydrate-binding motive is also located); whereas the rest of the protein is mainly composed of alpha helices (Figure 6). This bias in the secondary structure suggests that the beta sheets might form a structure that stabilizes this region.

## 4. Conclusions

The results presented here show that Vip3A proteins, and by extension other Vip3 proteins, are readily cleaved at a primary site by proteases rendering the 19–22 kDa and 62–66 kDa fragments. Despite the fact that the two fragments remain attached, the long-time exposure to proteases seems to eventually digest the small fragment. In contrast, the largest fragment is extremely stable to trypsin or MJ proteases. However, its susceptibility to SDS unfolding, along with the low efficacy of trypsin inhibitors to stop the proteolytic reaction, makes the SDS-PAGE analysis reveal secondary cleavage sites which give artefactual band patterns and, in some cases (at high concentration of MJ or trypsin), even the apparent complete degradation of the protein. The information provided here is useful for further biochemical and structural studies with the Vip3Aa and other Vip3 proteins, and may help explain some reproducibility problems faced when working with these type of proteins.

## 5. Materials and Methods

### 5.1. Vip3Aa Expression and Purification

The Vip3Aa protein was obtained from the vip3Aa16 gene fused to a six histidine-tail and cloned in *E. coli* [11]. Expression of the vip3Aa16 gene was achieved as described elsewhere [15], except for that the pre-culture was allowed to reach an OD of 1.2 before being transferred to the main culture medium, and that isopropyl-β-d-thiogalactopyranoside (IPTG) was added to the latter when it reached an OD of 0.4. After 5 h at 37 °C, the cells were harvested by centrifugation and then lysed [15]. After centrifugation at 17,000 *g*, the supernatant containing the Vip3Aa protein was collected and used for subsequent purification.

Affinity chromatography purification of Vip3Aa was carried out using His TrapTM FF crude columns (GE Healthcare Bio-Sciences AB, Uppsala, Sweden). The column was equilibrated with Phosphate-Buffered Saline solution (PBS, Roche, Germany) pH 7.4, with 10 mM of imidazole. The supernatant was then loaded onto the column and washed with PBS with 45 mM imidazole. The Vip3Aa protein was eluted with elution buffer (PBS containing 150 mM imidazole) and 1 mL fractions were collected in tubes containing 50 μL of 0.1 M EDTA. Fractions with a high protein concentration (as determined photometrically at 280 nm) were combined and dialyzed overnight against 20 mM Tris-HCl, 0.15 M NaCl, 5 mM EDTA, pH 8.6, or against 50 mM carbonate buffer, 5 mM EDTA, pH 10.5. The final concentration of Vip3Aa was determined either by densitometry after SDS-PAGE or by the Bradford’s method [40], using bovine serum albumin (BSA) as a standard.

### 5.2. Vip3A Proteolytic Processing

The Vip3Aa protein was subjected to different proteolysis treatments. Aliquots were taken out at desired times, mixed with SDS-PAGE loading buffer (10 μL sample with 5 μL loading buffer), heated at 99 °C for 10 min, snap frozen in liquid nitrogen, and stored at −20 °C until use. When trypsin inhibitors were used, the aliquots were mixed with trypsin inhibitors and either immediately or after a short incubation time (10 min at RT), the loading buffer was added to the samples to be processed for SDS-PAGE as usual. The loading buffer composition was 0.2 M Tris-HCl pH 6.8, 1 M sucrose, 5 mM EDTA, 0.1% bromophenol blue, 2.5% SDS, and 5% β-mercaptoethanol.

#### 5.2.1. Trypsin Treatments

The affinity-purified Vip3Aa protein was subjected to proteolytic activation with commercial trypsin (trypsin from bovine pancreas, SIGMA T8003, Sigma-Aldrich, St. Louis, MO, USA) in either Tris-HCl buffer (20 mM Tris-HCl, 0.15 M NaCl, 5 mM EDTA, pH 8.6) or carbonate buffer (50 mM carbonate buffer, 5 mM EDTA, pH 10.5). Vip3Aa was incubated with trypsin at either 1:100, 24:100, or 120:100 ratios (trypsin:Vip3A, *w*:*w*) at both 4 °C and 30 °C.

The irreversible inhibitors used to stop the trypsin action, were: PMSF (phenylmethanesulfonyl fluoride, from SIGMA), TLCK (Nα-tosyl-l-lysine chloromethyl ketone hydrochloride, from SIGMA), AEBSF protease inhibitor (from ThermoFisher, Waltham, MA, USA), and E64 (trans-epoxysuccinyl-l-leucylamido (4-guanidino) butane, from SIGMA). The denaturing agent used, urea, was added directly to the sample tubes to reach the concentration of 8 M.

#### 5.2.2. Midgut Juice (MJ) Treatment

MJ was obtained from *A. ipsilon* fifth instar larvae. For that purpose, 15 larvae were dissected and their peritrophic membranes, containing the food bolus, were pulled out and transferred into an ice-cold container, homogenized, and centrifuged for 10 min at 16,100 *g* at 4 °C. The supernatant was collected and centrifuged again for 10 additional min. The final supernatant was quickly distributed into small aliquots, snap frozen in liquid nitrogen, and stored at −80 °C. The protein content was quantified by the Bradford [40] method using BSA as a standard.

Vip3Aa was incubated with MJ at a ratio of 40:100 (MJ:Vip3A, *w*:*w*). The sample was incubated at 30 °C for 30 min.

### 5.3. MALDI TOF/TOF Analyses

The analyses were performed in a 5800 MALDI TOF/TOF (ABSciex) at the proteomics facility of the SCSIE (Servei Central de Suport a la Investigació Experimental), at the University of Valencia, Valencia, Spain. Protein fingerprinting was performed on tryptic fragments separated by SDS-PAGE.

To perform the molecular weight analyses, the Vip3Aa protein was first treated with trypsin (24:100 trypsin:Vip3A, *w*:*w*) and the mixture was incubated at 30 °C for 3 days. The analyses of the Vip3Aa molecular weight were performed after diluting the sample 1:2 in trifluoroethanol with SA as a matrix. The analyses were performed in a positive linear mode in a mass range of 10,000–100,000 *m*/*z*. The spectra were analyzed by the mMass software [41], Version 5.5.0.

### 5.4. Gel Filtration Chromatography

Gel filtration chromatography was performed with an ÄKTA explorer 100 chromatography system in a Superdex-75 10/300 GL column (GE Healthcare Life Sciences, Uppsala, Sweden) equilibrated and eluted with 20 mM Tris-HCl, 300 mM NaCl, pH 8.9, to a flow rate of 1 mL/min.

### 5.5. Toxicity Tests

The biological activity of the Vip3A samples was assessed with *A. ipsilon* first instar larvae. The *A. ipsilon* colony was established with insects obtained from Andermatt Biocontrol AG (Stahlermatten, Switzerland) which had been reared in the laboratory for more than 14 generations. The insects were reared on an artificial diet and maintained in a rearing chamber at 25 ± 3 °C, with 70 ± 5 RH and a 16/8 h light/dark photoperiod.

Surface contamination assays were carried out with a single larva in each 2 cm^2^ well in multiwell plates. The toxicity of full length Vip3Aa or the processed Vip3Aa was tested using protein concentrations of 40 and 65 ng/cm^2^ (which corresponds to the LC_90_ value extrapolated from published data [25]). For each replicate, 16 to 32 neonate larvae were used. Mortality was scored after 7 days. The larvae remaining in the first instar stage after 7 days were also recorded and added to the number of dead larvae to obtain the “functional mortality”.

### 5.6. Protein Structure Prediction Software

The secondary structure prediction was generated by the Geneious software, version 10.1.1. [42].

## Figures and Tables

**Table 1:** Toxicity of Vip3Aa before and after different trypsin treatments.

Vip3Aa Treatment	40 ng/cm^2^ Vip3Aa			65 ng/cm^2^ Vip3Aa		
	*n*	% Mortality	% Functional Mortality	*n*	% Mortality	% Functional Mortality
Untreated	2	71 ± 30	97 ± 4	3	81 ± 19	100
30 min trypsin-treated	2	67 ± 33	94 ± 8	2	94 ± 6	100
3–4 days trypsin-treated	2	66 ± 35	100	3	84 ± 16	100

Bioassays were performed with *A. ipsilon* neonates and the mortality scored after 7 days. Functional mortality is defined as the number of dead larvae plus first instar arrested larvae. Values represent the mean and standard error of n replicates. Mortality in the controls (just buffer or buffer with trypsin added) was always ≤10%.

## Supplementary Materials

The following are available online at www.mdpi.com/2072-6651/9/4/131/s1, Figure S1: Molecular mass determination by MALDI TOF/TOF of the 66 kDa polypeptide formed after treatment of the Vip3Aa protoxin with trypsin (24:100 trypsin:Vip3A, *w*:*w*) for 3 days.
