# Supplementary material for: Insights into the Structure of the Vip3Aa Insecticidal Protein by Protease Digestion Analysis
Source: Toxins (Basel). 2017 Apr 7;9(4):131. doi: 10.3390/toxins9040131 (PMC5408205; doi:10.3390/toxins9040131)
Supplement: Supplementary file 1 [file toxins-09-00131-s001.pdf]

Supplementary Materials: Insights into the Structure of the Vip3Aa Insecticidal Protein by Protease Digestion Analysis

Yolanda Bel, Núria Banyuls, Maissa Chakroun, Baltasar Escriche, Juan Ferré*

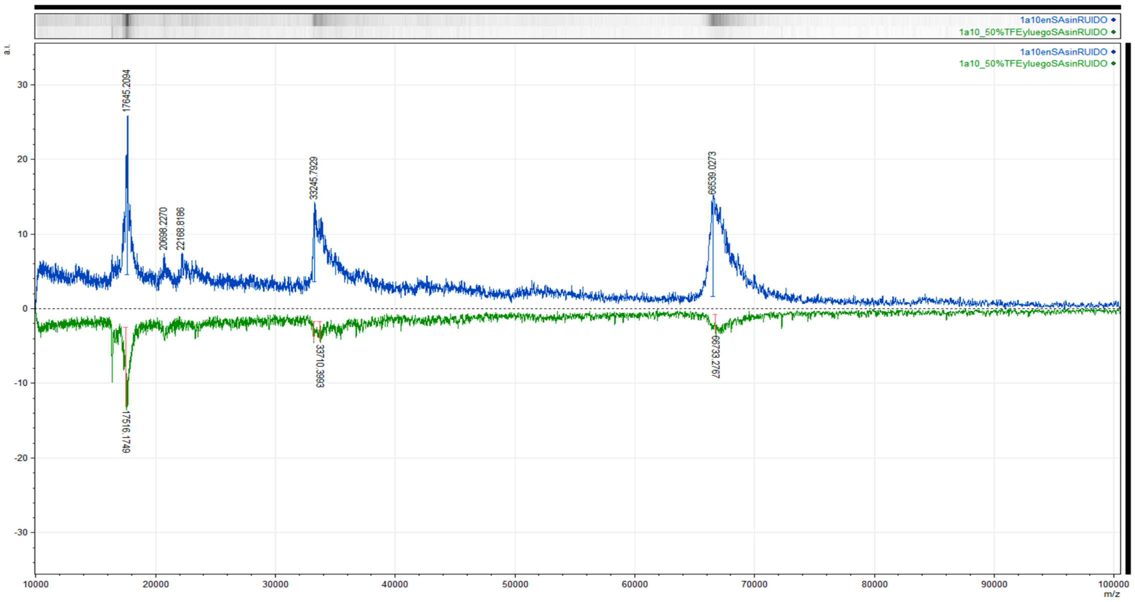

Figure S1. Molecular mass determination by MALDI TOF/TOF of the 66 kDa polypeptide formed after treatment of the Vip3Aa protoxin with trypsin (24:100 trypsin:Vip3A, w:w) for 3 days.
